# mAbXcite: a novel immunotherapy platform that initiates a robust anti-cancer immune response by recruiting and activating neutrophils

**DOI:** 10.1186/2051-1426-2-S3-P262

**Published:** 2014-11-06

**Authors:** Ifat Rubin-Bejerano, Isabelle Sansal-Castellano, Mark Carlson, Gabriel Reznik, James Siedlecki, John Kane, Zuzana Dostalova, Hua Miao

**Affiliations:** 1 ImmuneXcite Inc., USA

## 

The recent successes in the immunotherapy field demonstrate that a successful immune response to cancer can lead to meaningful anti-tumor responses. Antibodies blocking immune checkpoint molecules (e.g. CTLA4, and PD-1) lead to effective T cell responses especially in tumors that are immune active.

Immune cells that have not received as much attention in cancer immunotherapy are neutrophils. Neutrophils are the most abundant white blood cells, which protect us from microbial infections. They are the first line of innate defense: they arrive early on in the course of infection, phagocytose, and release the content of their granules, including reactive oxygen species and enzymes. Neutrophils also communicate with other immune cells, such as macrophages, dendritic cells, and T cells, by releasing cytokines and chemokines.

There has long been a call to recruit these professional killers to fight cancer. However, an approach that mediates the recruitment of neutrophils has to lead to acute as opposed to chronic inflammation to overcome the suppressive environment that tumors surround themselves with.

We have developed a novel immunotherapy platform technology, termed mAbXcite, which directs and activates neutrophils to kill cancer cells in a targeted manner. The targeting is achieved by using monoclonal antibodies that are chemically linked to beta-1,6-glucan, a saccharide found on the cell walls of fungal species which recruits and activates neutrophils. The resulting mAb construct attracts mediators of acute inflammation, notably neutrophils, leading to rapid destruction of cancer cells.

We will present proof-of concept of the mAbXcite technology using two validated antibodies, the anti-Her2 Trastuzumab and the anti-EGFR Cetuximab. In preclinical xenograft models, these mAbXcite constructs demonstrate significantly greater efficacy than the original antibody in resistant tumor models. Furthermore, some mice show complete regressions and do not grow tumors upon rechallenge, suggesting a long term immune response. We will also provide pharmacodynamics results, primarily neutrophil infiltration by histology and live imaging, as well as pharmacokinetics results, demonstrating that the linked oligosaccharide is stable and is not affecting the half-life of the antibodies.

This novel immunotherapy platform is suitable for peripheral membrane targets that are overexpressed on tumors, and does not require antibody internalization. The unique mechanism of action of recruiting and activating neutrophils against cancer could be synergistic with other immunotherapeutic approaches.

